# Human Pressures on Natural Reserves in Yunnan Province and Management Implications

**DOI:** 10.1038/s41598-018-21654-w

**Published:** 2018-02-19

**Authors:** Cheng Qiu, Jinming Hu, Feiling Yang, Feng Liu, Xinwang Li

**Affiliations:** 1 Institute of International Rivers and Eco-security, Yunnan University, Kunming, Yunnan 650500 China; 2 School of Geography and Land Engineering, Yuxi Normal University, Yuxi, Yunnan 653100 China; 3 Yunnan Key Laboratory of International Rivers and Transboundary Ecosecurity, Yunnan University, Kunming, Yunnan 650500 China; 4 Collaborative Innovation Center for Territorial Sovereignty and Maritime Rights, Wuhan, Hubei 430072 China

## Abstract

The analysis of status and major sources of human pressures on natural reserves (NRs) is important for optimizing their management. This study selected population density, gross domestic product (GDP) density and areal percentage of human land use to reveal the human pressures of national and provincial NRs (NNRs and PNRs) in Yunnan Province, China. We calculated three types of internal and external human pressure index (HPI) and comprehensive HPI (CHPI) for NRs. Human pressures on most of NRs were slight and light, indicating that most of NRs were well protected. Human pressures on PNRs were higher than on NNRs; with respect to five types of NRs, geological relict NRs were facing the highest human pressures, followed by wetland ecosystem NRs. Land use and population density were the main human pressures on these NRs. Yunnan Province should put the highest emphasis on three NNRs and two Ramsar site PNRs with severe CHPI, secondly pay attention to eight conservation-oriented PNRs with extreme or severe CHPI. It’s urgent for Yunnan to implement scientific policies and measures to reduce land use and population density pressures of NRs, especially with severe and extreme CHPI, by transforming internal land use and/or implementing residents’ eco-migration.

## Introduction

Biodiversity loss is accelerating^1^, and increasing human activities that contribute to the degradation of natural ecological systems are the main causes^2–4^. *In situ* protected area systems, the core of which is the natural reserve (NR), are the most direct and effective modes of biodiversity conservation^5–7^, but established protected areas have encountered increasing human disturbances and pressures, directly influencing their management effectiveness^8,9^. The analysis of human pressures on protected areas is of great importance for recognizing the effectiveness of protected areas, as well as developing and optimizing management strategies and policies^10,11^. Moreover, where human disturbance is intense, the conservation cost is high for systematic conservation planning^12,13^. Therefore, the design, development, management and expansion of existing protected areas should consider human pressures^14^.

Yunnan Province is one of the most biodiverse regions in the world and receives considerable research attention^15–17^. However, human activities during the past several decades have posed a series of serious threats to biodiversity in Yunnan^18,19^. Yunnan is now in a period of accelerating industrialization and urbanization, and most NRs were in underdeveloped areas facing the dual pressures of biodiversity conservation and economic development^17^. In 2013, China proposed the Belt and Road Initiative (BRI, i.e., a Silk Road Economic Belt and a 21st Century Maritime Silk Road), which is aimed at building a trade and infrastructure network connecting China with Southeast and South Asia, Central and West Asia, Europe and Africa along ancient trade routes. With the implementation of the BRI as well as Yunnan Provincial regional development strategy, Yunnan plans to carry out large-scale construction of infrastructure (e.g. Baoshan-Lushui railway, Lincang-Pu’er railway and Dali-Lincang express way, and so on). These human activities will bring great human pressures to the management of the NRs in Yunnan Province and regional biodiversity conservation. Meanwhile, the Yunnan Province Biodiversity Conservation Strategy and Action Plan (2012–2030) proposed to optimize the spatial pattern of NNRs and PNRs and strengthen the development and management of NRs in the priority areas. Yunnan Province 13^th^ Five-year Plan for Economy & Social Development states that eco-migration policy will be gradually implemented to remove the people residing inside the NRs and reduce population pressure on NRs. These urgent demands require us to reveal the status of ever-increasing human pressures and the major pressure type on NRs in Yunnan Province.

As we examined the basic data of all NRs in Yunnan Province, we found only NNRs and PNRs have clear geographical boundaries. The Yunnan Province Biodiversity Conservation Strategy and Action Plan (2012–2030) proposed to optimize the pattern and promote management effectiveness of NNRs and PNRs. Hence, this study selected all 20 NNRs and 38 PNRs (totally 58 NRs) in Yunnan Province to reveal their human pressures. Considering data availability and comparability, we selected three types of human pressures, i.e., population density, GDP density and areal percentage of human land use, which were often used as the main proxies for analysing human pressures on NRs^11,20–25^. We calculated internal (within each NR) and external (2 km external buffer region outside each NR, the same below) population density pressure index (PDPI), GDP density pressure index (GDPI) and human land use pressure index (HLUPI), and then internal and external CHPI of all 58 NRs in Yunnan Province. Using Jenks natural breaks method to reduce the variance within each HPI class, we reclassified the internal and external individual HPI (PDPI, GDPI, HLUPI) and comprehensive HPI respectively in five levels: slight, light, moderate, severe, and extreme. Analysing the internal and external individual and comprehensive human pressures, we identified the NRs with severe and extreme internal CHPI (ICHPI) and/or external CHPI (ECHPI) and their main pressure types.

## Results

### Three types of human pressures on NRs

**Figure 1:**
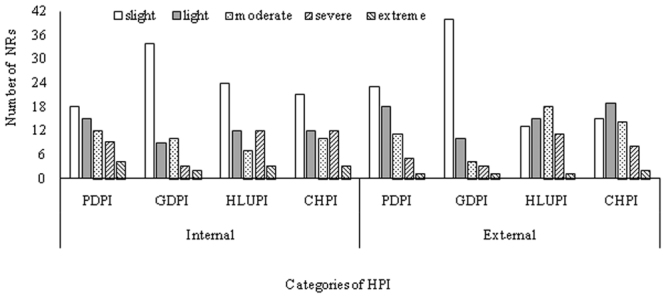
Number of NRs in Yunnan Province by HPI levels.

Figure 1 and Table 1 showed the variation of internal and external HPI and CHPI of 58 NRs in Yunnan Province. The number of NRs with slight and light HPI (whether internal or external) was significant higher than that of NRs with severe and extreme HPI (Fig. 1), indicating that most of 58 NRs in Yunnan Province have been well protected. The mean value of internal individual and comprehensive HPI of all 58 NRs was significantly (*P* < 0.05 or 0.01) lower than the corresponding external value (Table 1). The Coefficient of Variation (CV) of GDPI was the highest, followed by PDPI and HLUPI (Table 1). The number of NRs with severe and extreme internal GDPI, PDPI and HLUPI accounted for 8.62%, 22.41% and 25.86%, respectively; the number of NRs with severe and extreme external GDPI, PDPI and HLUPI accounted for 6.90%, 10.34% and 20.69%, respectively.

**Table 1:** Human pressure difference of NRs in Yunnan Province.

Internal/External	Characteristic values of HPI	PDPI	GDPI	HLUPI	CHPI
internal	Minimum	0.00	0.01	0.00	0.00
	Mean	0.71	0.80	0.80	0.77
	Maximum	3.67	10.83	3.77	5.13
	Coefficient of Variation	1.27	2.24	1.00	1.33
external	Minimum	0.01	0.01	0.03	0.01
	Mean	1.29	1.20	1.20	1.23
	Maximum	18.38	17.39	3.48	9.86
	Coefficient of Variation	1.91	2.23	0.56	1.38
Significance of mean value difference	P-value	0.001^**^	0.036^*^	0.000^**^	0.002^**^

Note: * indicates a significant difference at the 5% level; ** indicates a significant difference at the 1% level. PDPI (population density pressure index), GDPI (GDP density pressure index), HLUPI (human land use pressure index), CHPI (comprehensive human pressure index), the same below.

Table 2 showed that the mean value of each internal HPI of PNRs was higher than that of NNRs, among which the mean values of internal GDPI and HLUPI of PNRs were significant higher than these of NNRs; but the mean values of all external HPI showed no significant differences between the NNRs and PNRs. 15 (internal) and 12 (external) NRs were facing severe and extreme HLUPI, and 13 (internal) and 6 (external) NRs were facing severe and extreme PDPI (Fig. 1). In terms of three human pressure types, human land use was the major pressure on these 58 NRs in Yunnan Province, followed by population density and GDP density pressures.

**Table 2:** HPI differences between NNRs and PNRs in Yunnan Province.

Internal/External	levels	Characteristic values of HPI	PDPI	GDPI	HLUPI	CHPI
internal	National	Minimum	0.02	0.01	0.01	0.03
		Mean	0.49	0.39	0.51	0.46
		Maximum	2.62	3.15	1.72	1.87
	Provincial	Minimum	0.00	0.01	0.00	0.00
		Mean	0.83	1.01	0.96	0.93
		Maximum	3.67	10.83	3.77	5.13
	Significance of mean value difference	P-value	0.109	0.041^*^	0.030^*^	0.067
external	National	Minimum	0.16	0.04	0.18	0.13
		Mean	1.06	1.32	1.06	1.15
		Maximum	5.31	17.39	2.27	8.69
	Provincial	Minimum	0.01	0.01	0.03	0.01
		Mean	1.41	1.14	1.28	1.28
		Maximum	18.38	7.15	3.48	9.86
	Significance of mean value difference	P-value	0.731	0.185	0.273	0.273

Note: * indicates a significant difference at the 5% level.

### Comprehensive human pressures on NRs

Table 1 showed that the mean value of NRs’ ICHPI was significantly (*P* < 0.01) lower than that of NRs’ ECHPI. The NRs with severe and extreme ICHPI (ECHPI) accounted for 25.86% (17.24%) of the total 58 NRs. Generally, the order of 58 NRs’ ICHPI was similar to that of 58 NRs’ ECHPI (*see the* Supplementary Table S1); that is to say, if the NR was facing low (high) internal comprehensive human pressure, then the NR was also facing low (high) external comprehensive human pressure. However, there were some NRs whose ICHPI and ECHPI order has large difference. For instance, the ICHPI (ECHPI) order of Yongde Snow Mountain, Longling Xiaoheishan and Napa Lake NRs was 2 (25), 16 (44) and 32 (55), respectively. In contrast, the ICHPI (ECHPI) order of Tengchong Beihai Wetland, Guangnan Babao and Menglian Mountain NRs was 58 (37), 52 (29) and 44 (18), respectively.

**Figure 2:**
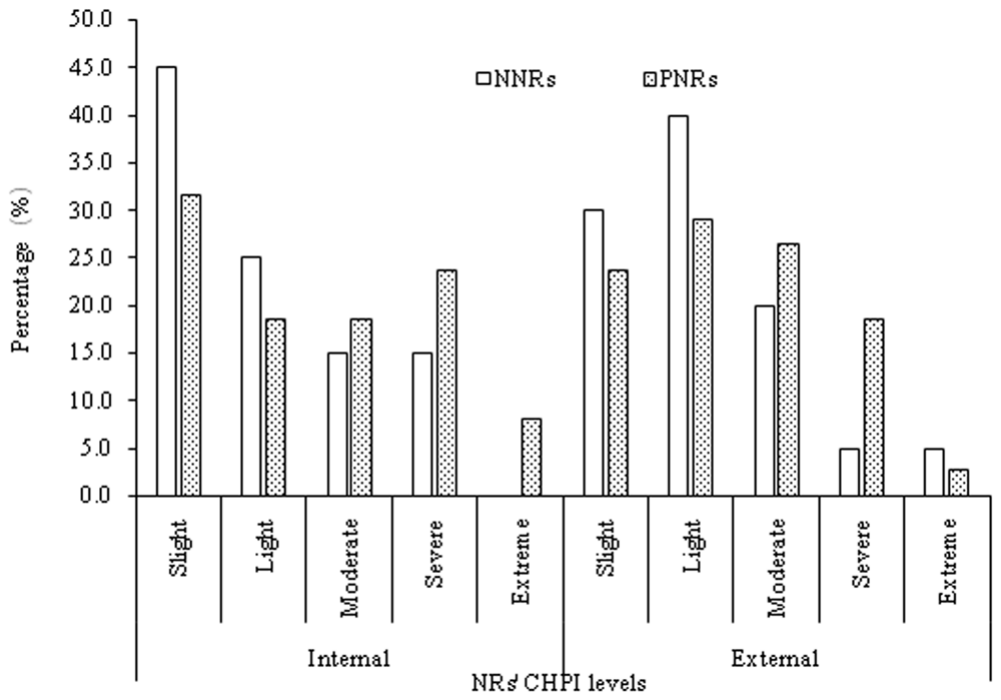
The number percentages of NNRs or PNRs in Yunnan Province by ICHPI and ECHPI levels.

The NNRs with slight and light ICHPI accounted for 70% and the NNRs with severe and extreme ICHPI accounted for 15% of all 20 NNRs; correspondingly, the PNRs with slight and light ICHPI, and severe and extreme ICHPI accounted for 50% and 31.58% of all 38 PNRs, respectively (Fig. 2). Similarly, the NNRs with slight and light ECHPI accounted for 70% and the NNRs with severe and extreme ECHPI accounted for 10% of all NNRs; correspondingly, the PNRs with slight and light ECHPI, and severe and extreme ECHPI accounted for 52.63% and 21.05% of all 38 PNRs, respectively (Fig. 2). Thus, the comprehensive human pressures on the PNRs were higher than those on the NNRs. But Table 2 also showed the ICHPI and ECHPI of the PNRs were not significantly higher than those of NNRs.

### The human pressures on different NR types

Table 3 showed internal and external human pressure differences among five NR types. Internal PDPI, GDPI and CHPI had significant differences (*P* < 0.01) among the five NR types, but internal HLUPI had no significant difference. External PDPI had significant difference (*P* < 0.05) among the five NR types, but other three external HPI had no significant differences. In terms of five NR types, geological relict NRs had the highest mean values of internal and external PDPI, GDPI and CHPI, followed by wetland ecosystem NRs; wild plant NRs and wild animal NRs had close and low individual HPI and CHPI; while forest ecosystem NRs had the lowest mean values of individual HPI and CHPI.

**Table 3:** HPI difference among the five types of NRs in Yunnan Province.

Internal/External	NRs’ types (number)	PDPI	GDPI	HLUPI	CHPI
internal	Geological relicts (3)	2.4959	3.6026	1.3244	2.5600
	Wild plants (4)	0.8761	0.2041	1.4801	0.8076
	Wild animals (8)	0.8073	0.6327	1.1977	0.8537
	Wetland ecosystems (10)	1.3348	2.2606	0.9072	1.5403
	Forest ecosystems (33)	0.3219	0.2131	0.5415	0.3443
	P-value	0.005^**^	0.004^**^	0.152	0.005^**^
external	Geological relicts (3)	7.4427	3.7244	1.5087	4.5132
	Wild plants (4)	0.6869	0.3446	1.4900	0.7883
	Wild animals (8)	0.7943	0.6580	1.5159	0.9452
	Wetland ecosystems (10)	1.8894	3.7103	1.2239	2.3419
	Forest ecosystems (33)	0.7350	0.4472	1.0532	0.7220
	P-value	0.028^*^	0.149	0.340	0.057

Note: number in bracket is the original number of specific NR type among all 20 NNRs and 38 PNRs in Yunnan Province.

*Indicates a significant difference at the 5% level; ** indicates a significant difference at the 1% level.

**Figure 3:**
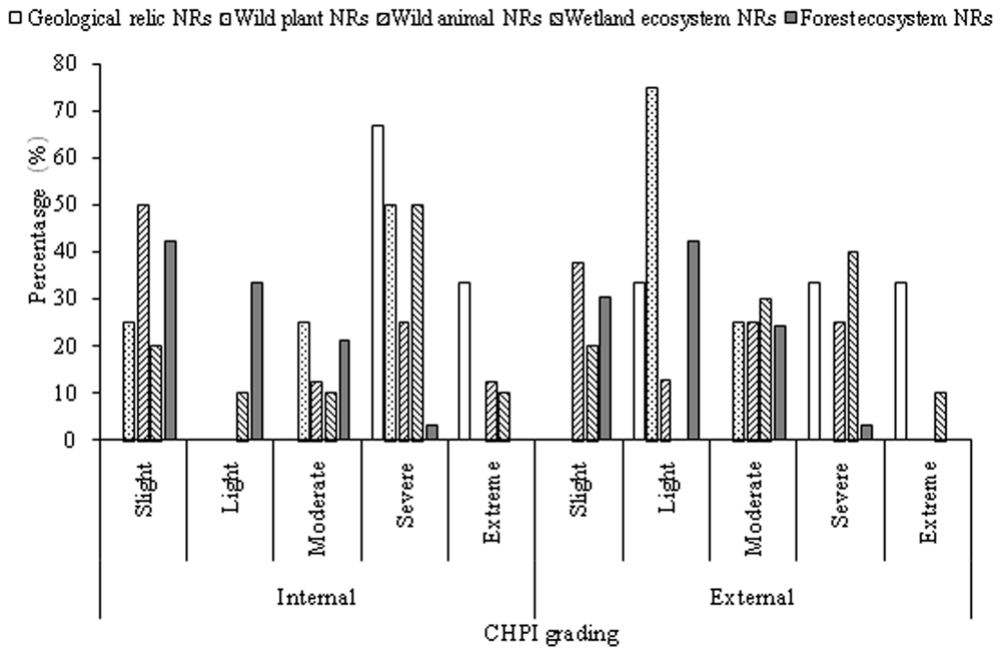
The number percentages of each type of NRs in Yunnan Province by ICHPI and ECHPI levels.

Table 4 showed the number of each type of NRs whose individual HPI and CHPI were severe and extreme. Whether internal or external (Tables 3 and 4), population density and GDP density pressures on wetland and geological relict NRs were higher than those of other three types of NRs, but human land use pressure among the five NR types showed no significant difference. Table 3, Table 4 and Fig. 3 showed that the mean values of ICHPI and ECHPI of geological relict NRs were the highest, and three geological relict NRs had severe or extreme ECHPI; while those of forest ecosystem NRs were the lowest and only Pearl River head source PNR had severe ICHPI and ECHPI.

**Table 4:** Number of each type of NRs in Yunnan Province with severe and extreme HPI.

Internal/External	NRs types (number)	PDPI	GDPI	HLUPI	CHPI
Internal	Geological relicts (3)	3	1	2	3
	Wild plants (4)	1	0	3	2
	Wild animals (8)	2	1	3	3
	Wetland ecosystems (10)	5	3	4	6
	Forest ecosystems (33)	2	0	3	1
	Total	13	5	15	15
External	Geological relicts (3)	2	1	2	2
	Wild plants (4)	0	0	1	0
	Wild animals (8)	0	0	3	2
	Wetland ecosystems (10)	4	3	2	5
	Forest ecosystems (33)	0	0	4	1
	Total	6	4	12	10

Note: number in bracket is the original number of specific NR type among all 20 NNRs and 38 PNRs in Yunnan Province.

## Discussion

Substantial population growth, rapid economic development and drastic land-use and land-cover change (LUCC) have been the main sources of human pressures that decreased effective conservation and management of on-site regional and global biodiversity protected areas^9,21,26–31^. Analysis of human pressures of existing NRs can provide valuable information for optimizing regional protected areas system^10,11,20^. The internal and external human pressures on most NNRs and PNRs in Yunnan Province were slight or light (Figs 1 and 2), which indicated that these NRs were well protected^32^. However, among these 58 NRs, 25.9% had severe or extreme ICHPI, and 17.2% had severe or extreme ECHPI. Identification of the NRs with severe or extreme ICHPI or ECHPI, as well as the main sources of human pressures, offers a strong support for their management optimization. We reclassified these 58 NRs in two groups: species or ecosystems conservation-oriented NRs and geologic relict NRs. Ordering the two groups separately by NR levels (from national to provincial), and descending ICHPI and ECHPI values, we identified the NRs with severe or extreme ICHPI or ECHPI, and their main sources of human pressures (Table 5).

**Table 5:** The priority NRs *** in Yunnan Province.

NO.	Natural reserves	Grade	Type	ICHPI level	ICHPI rank^*^	Internal main pressures^**^	ECHPI level	ECHPI rank^*^	External main pressures^**^
1	Dashanbao	national	wetland	severe	53	PDP/HLUP	moderate	48	PDP
2	Cang Mountain and Erhai Lake	national	wetland/forest	severe	50	GDP	extreme	57	GDP/PDP /HLUP
3	Huize Black-necked Cranes	national	animal	severe	49	PDP/HLUP	severe	51	
4	Tengchong Beihai Wetland	provincial	wetland	extreme	58	PDP/GDP	moderate	37	
5	Jianshui Swallow Cave	provincial	Animal	extreme	56	PDP/HLUP/GDP	severe	49	HLUP
6	Lashi Lake	provincial	wetland	severe	55	PDP/GDP/HLUP	severe	56	PDP/GDP
7	Qiubei Puzhehei	provincial	wetland	severe	51	PDP/HLUP	severe	50	
8	Jian Lake	provincial	wetland	severe	48	PDP/HLUP	severe	53	PDP/HLUP
9	Pear River’s Source	provincial	forest	severe	47	PP/LUP	severe	52	HLUP
10	Jiache	provincial	plant	severe	46	PDP/HLUP	moderate	45	
11	Xundian Black-necked Cranes	provincial	animal	severe	45	HLUP	moderate	36	HLUP
12	Menglian Mountain	provincial	plant	severe	44	HLUP	light	18	
13	Napa Lake	provincial	wetland	light	32		severe	55	GDP
14	Jinning Meishucun	provincial	geological relicts	extreme	57	PDP/GDP/HLUP	extreme	58	PDP/GDP/HLUP
15	Chengjiang Fossil Site	provincial	geological relicts	severe	54	PDP/HLUP	severe	54	PDP/HLUP
16	Guangnan Babao	provincial	geological relicts	severe	52	PDP	light	29	

^*^The total number of NRs in this study was 58; the larger the rank of ICHPI and ECHPI, the higher the corresponding HPI.

^**^The severe or extreme internal and external main pressures were determined according to Supplementary Table S1.

***Priority orders: We first reclassified the 58 NRs in two groups: species or ecosystems conservation-oriented NRs and geologic relict NRs. Ordering the two groups separately by national to provincial levels, descending ICHPI and ECHPI values, we identified the NRs with severe or extreme ICHPI or ECHPI.

PDP (population density pressure), GDP (GDP density pressure), HLUP (human land use pressure).

### Land use pressures

LUCC has become one of the most direct and primary drivers of global biodiversity loss^3,33–35^. During the past several decades, drastic LUCC in Yunnan Province^31,36–39^ have exacerbated regional biodiversity loss^16,40,41^ and threatened the effective management of existing NRs^39,42^. Table 1 showed the variations of internal and external human land use pressure on NRs were minimal. Whether internal or external, human land use pressure had no significant difference among the five NR types (Table 3). Each of the five NR types had one to four NRs with severe and extreme HLUPI, and the NRs with severe and extreme internal (external) land use pressure accounted for 25.86% (20.7%). Viewing from Table 5, human land use was the major human pressure source for these NRs with severe and extreme ICHPI or ECHPI. Most importantly, Huize and Dashanbao Black-necked Crane NNRs were also facing severe internal and moderate external human land use pressure.

Among these NRs with severe or extreme HLUPI, average areal percentage of internal and external farmlands was 38.26% and 41.53%, respectively, and average areal percentage of the construction lands was only 1.04% and 2.26%, respectively. Areal percentage of internal (external) farmlands of Jianshui Swallow Cave NR reached the highest to 80%, followed by Xundian Black-necked Crane NR. Both internal and external farmlands area of the Pearl River Source NR were the largest (totally more than 100 thousand hm^2^), followed by Guanyin Mountain NR. Areal percentage of construction lands of these NRs with severe or extreme HLUPI was usually low. Among the three NNRs in Table 5, areal percentages of internal arable lands in Huize and Dashanbao Black-necked Cranes NNRs reached 39.59% and 36.53%, respectively, and areal percentage of external arable land in Cang Mountain and Erhai Lake NNR reached 42.3%.

O’Connell-Rodwell *et al*.^43^ reported that farmland expansion was a major driver of global biodiversity loss and often caused conflicts between regional biodiversity conservation and socio-economic development. High human land use pressure affected the management effectiveness and even resulted in a certain degree of NRs’ degradation. China’s 13^th^ Five-Year Plan for Ecological & Environmental Protection (2016–2020) proposed to implement mandatory environmental protection in prohibited development areas and strictly prohibit relevant development activities that are inconsistent with function orientation of various functional zones. This study revealed that human land use was the main human pressure on most NNRs and PNRs in Yunnan Province, and farmland was the main source of land use pressure. Hence, it’s urgent for Yunnan to relieve the farmland pressure on the following NRs: Huize and Dashanbao Black-necked Crane NNRs (both with severe internal HLUPI), Lashi Lake Ramsar site PNR (with severe internal HLUPI), and other PNRs with both internal and external severe or extreme HLUPI (Jianshui Swallow Cave, Xundian Black-necked Crane, Chengjiang Fossil Site, Mojiang Xiqi *Alsophila spinulosa*, Guanyin Mountain, Pearl River Source, Jian Lake and Jinning Meishucun PNRs).

### Population density pressures

Regional population density (or size) is often considered to be primary cause of the decline in biodiversity and ecosystem services^44,45^. Population growth sometimes increased regional biodiversity protection cost^46,47^, and decreased the area^48,49^ and effectiveness of existing protected areas^9,27^. Most NRs in China were in relatively remote areas. With rapid rural economy development and ever-increasing of human activities (e.g. highways and railways construction), most NRs in China are facing high population pressure^16,29^. High population pressure usually leads to high human land use and/or GDP density pressures, which has caused the decline of the NRs’ management effectiveness.

From 1958 to 2010, population in Yunnan Province had increased from about 19 Million to 46 Million^50^. This substantial population growth had accelerated biodiversity loss in Yunnan Province^16,40,41^, which brought about tremendous pressures on the development and effective management of the NRs in Yunnan. Tables 3 and 4 showed that population density pressure on geological relict and wetland ecosystem NRs was relatively high. For most NRs with severe or extreme ICHPI or ECHPI (Table 5), population density was also one of the major human pressure sources. In terms of the population density of these 58 NRs in Yunnan Province, the internal highest and lowest was about 366 and 131 people/km^2^, respectively, and the external highest and lowest was about 1833 and 253 people/km^2^, respectively. Among the 13 NRs with severe or extreme internal PDPI, Dashanbao Black-necked Crane NNR had extreme internal and severe external PDPI, and Huize Black-necked Crane NNR had severe internal and moderate external PDPI.

China’s 13^th^ Five-Year Plan for Ecological and Environmental Protection (2016–2020) pointed out that government will implement eco-migration policy in prohibited development areas and gradually remove the people residing in the core and buffer areas of the NRs. This is also one of the top priorities for management optimization of the NRs in Yunnan Province. Eco-migration plan and measures need to be systematically made and implemented gradually for 13 NRs with severe or extreme internal PDPI, among which the most urgent includes Dashanbao, and Huize Black-necked Crane NNRs and Yao Mountain NNR.

### GDP density pressures

GDP density pressure on these 58 NRs, mostly located in remote and underdeveloped areas in Yunnan Province^17,42^, was low. Only a few NRs, i.e., Tengchong Beihai Wetland PNR, Jinning Meishucun PNR, Lashi Lake PNR, Jianshui Swallow Cave PNR, and Cang Mountain and Erhai Lake NNR (Table 5), were facing severe GDP density pressure due to rapid socio-economic development. In terms of NRs’ types, wetland ecosystem NRs were facing relatively severe or extreme GDP density pressure. It’s urgent to regulate the negative impacts of disorder or excessive economic development (e.g. disorder tourism activities) upon these NRs with severe or extreme GDPI. However, for those NRs located in underdeveloped areas, low inputs inevitably limited their infrastructure construction and management capability promotion^30,32,42^, which was one of the major obstacles to improve the function of the NRs in Yunnan Province.

### Optimization priorities

Most NRs in Yunnan Province were in the impoverished or underdeveloped areas, where were facing the pressures of developing regional economy and conserving regional biodiversity^17^. Population density and human land use were the main and usually concomitant pressures on most NRs in Yunnan Province (Table 5). Under this context, Yunnan Province needs to issue scientific policies, measures and implementation schedules to transform human land use or/and remove the people residing in relevant NRs. Firstly, Yunnan should put the highest emphasis on three NNRs with severe ICHPI and two Ramsar site PNRs with severe ECHPI, i.e., Dashanbao Black-necked NNR, Cang Mountain and Erhai Lake NNR, Huize Black-necked Crane NNR, Lashi Lake and Napa Lake PNRs. Secondly, attention should be paid to eight species or ecosystem conservation-oriented PNRs with extreme or severe ICHPI, i.e., Tengchong Beihai Wetland, Jianshui Swallow Cave, Qiubei Puzhehei, Jian Lake, Pearl River Source, Jiache, Xundian Black-necked Crane and Menglian Mountain PNRs. While, we need further investigation of the human pressure status in the core and buffer areas of these 13 identified NRs, providing scientific decision-making supports to transform their internal human land use and/or implement their eco-migration.

## Conclusions

Human pressures on most of 58 NRs in Yunnan Province were slight and light, indicating that these NRs were well protected. But some NNRs and PNRs were still facing severe or extreme human pressures. More PNRs than NNRs were facing severe or extreme internal human land use and population density pressures, and severe or extreme external land use pressure. In terms of NRs types, geological relict NRs were facing the highest human pressures, followed in sequence by wetland ecosystem NRs, wild animal NRs, wild plant NRs and forest ecosystem NRs.

Human land use and population density were the main human pressures on these 58 NRs in Yunnan Province. Human land use pressure had no significant difference among the five NRs types, while population density and GDP density pressures on geological relict and wetland NRs were higher than those on other three types of NRs. Farmland use was the main source of human land use pressure.

Yunnan should firstly put the highest emphasis on three NNRs with severe ICHPI and two Ramsar site PNRs with severe ECHPI, and secondly pay attention to eight species or ecosystem conservation-oriented PNRs with extreme or severe ICHPI. It’s urgent for Yunnan Province to issue scientific policies, measures, and implementation schedules to transform human land use or/and remove the people residing in relevant NRs.

## Methods

### Spatial database of NRs

Yunnan Province Biodiversity Conservation Strategy and Action Plan (2012–2030) proposed to optimize the spatial pattern and promote the management effectiveness of NNRs and PNRs, whose area accounted for 77.50% of the total area of all NRs in Yunnan in 2015 (http://www.ynepb.gov.cn/zrst/zrbhq/201603/t20160321_150799.html). We examined the basic geographical data of all NRs in Yunnan Province and found only NNRs and PNRs have clear geographical boundaries. Hence, this study selected all 20 NNRs and 38 PNRs (totally 58 NRs) to reveal their human pressures. We collected the overall plans, annual reports and other data of all NNRs and PNRs in Yunnan Province to obtain their boundary maps. Through spatial registration and digitization, we obtained the vector data of each NR’s boundary and generated the spatial attribute database of all these 58 NRs (name, area, type, level, etc.).

### Human pressure data

Human pressures on the environment, commonly referred to as threats to biodiversity, are the actions taken by humans with the potential to harm nature^51^. The greater the number and intensity of human pressures in an area the more negative the prospects of biodiversity or of the habitat type^52^. Considering data availability and comparability, we selected three types of human pressures that are commonly used^11,20–25,51^, i.e., population density, GDP density and areal percentage of human land use (including farmlands and construction lands), to analyse human pressures on NRs in Yunnan Province. The data of population density, GDP density and land use in Yunnan Province in 2010 (the same below) were provided by the Data Center for Resources and Environmental Sciences, Chinese Academy of Sciences (RESDC; http://www.resdc.cn). Population density and GDP density are 1 km × 1 km resolution grid files, and land use data is 30 m × 30 m resolution grid files.

### Human pressure index calculation

Using ArcMap 10.2, we cut the original grid data files of population density, GDP density and land use in Yunnan Province by each NR’s boundary vector data to obtain each NR’s internal population density, GDP density and land use. We extracted the patches of farmlands and construction lands (as human land use) within each of these 58 NRs. Then we calculated each NR’s internal population, GDP and human land use area. After that, dividing each NR’s internal population, GDP and human land use area by each NR’s area, we obtained original internal population density pressure index (PDPI), GDP density pressure index (GDPI) and human land use pressure index (HLUPI) of each NR.

We generated a 2 km width external buffer region (EBR) for each of these 58 NRs. We obtained the inner boundary (i.e., the NR’s boundary) and outer boundary of each NR’s EBR. We calculated each NR’s EBR area. We used these inner and outer boundaries of each NR’s EBR to cut the original grid data files of population density, GDP density and land use in Yunnan Province to obtain each EBR’s population density, GDP density and land use. Repeating the similar processes, we obtained original external PDPI, GDPI and HLUPI of each NR.

We normalized the original internal and external PDPI, GDPI and HLUPI of each NR by using Mean Value Method (Equations (1) and (2)).

1 $$IHP{I}_{ij}=i{x}_{ij}/\overline{{x}_{j}}$$

2 $$EHP{I}_{ij}=e{x}_{ij}/\overline{{x}_{j}}\,$$

where *i* is the number of NRs (*i* = 1–58); *j* is the human pressure type (*j* = 1–3, indicating population density, GDP density and human land use pressures, respectively); $i{x}_{ij}\,(e{x}_{ij}$) is the original internal (external) *HPI* of human pressure type *j* for each NR *i*; $\overline{\,{x}_{j}}\,\,$is the mean values of original internal (external) *HPI* of human pressure type *j*; $\,IHP{I}_{ij}$($EHP{I}_{ij})\,\,$is the internal (external) *HPI* of human pressure type *j* of each NR *i*.

Finally, we calculated each NR’s ICHPI and ECHPI using Equations (3) and (4).

3 $$ICHP{I}_{i}=\sum _{j=1}^{3}IHP{I}_{ij}\ast {W}_{j}$$

4 $$ECHP{I}_{i}=\sum _{j=1}^{3}EHP{I}_{ij}\ast {W}_{j}$$

where *i* and *j* are the same as the equations 1 and 2; $ICHP{I}_{i}$ and ${\rm{E}}CHP{I}_{i}$are internal and external CHPI of NR *i*, respectively; $IHP{I}_{ij}$and ${\rm{E}}HP{I}_{ij}$are the same as equations 1 and 2; *W*_*j*_ (0.376, 0.349 and 0.275, respectively) is the weight of $IHP{I}_{ij}$ and $EHP{I}_{ij}$, calculated by principal component analysis via using IBM SPSS Statistics 22.0.

### Reclassification of HPI

Using Jenks natural breaks method, we reclassified each internal HPI (*IHPI*_*ij*_, *ICHPI*_*i*_) and external HPI ($EHP{I}_{ij}$, *ECHPI*_*ij*_) separately in five levels: slight, light, moderate, severe, and extreme (*see the* Supplementary Table S2) and then conducted following analysis.

### Mean comparison and test

Independent samples test (Mann-Whitney U test) was conducted to examine the significance of difference between the mean values of NR’s internal and external HPI (Table 1), between the mean values of NNRs’ and PNRs’ internal and external HPI (Table 2). Multiple independent-sample tests (Kruskal-Wallis test) were conducted to examine the significance of difference of the mean value of internal (external) HPI among five types of NRs (Table 3). We used IBM SPSS Statistics 22.0 to perform statistical analyses in this study.

## Electronic supplementary material

Supplementary Tables

## References

[1] Barnosky AD (2011). Has the earth’s sixth mass extinction already arrived?. Nature.

[2] Dirzo R, Raven PH (2003). Global state of biodiversity and loss. Annu. Rev. Environ. Resour..

[3] Millennium Ecosystem Assessment (MEA). *Ecosystems and human well-being: synthesis* (Island Press, 2005).

[4] Verones F, Moran D, Stadler K, Kanemoto K, Wood R (2017). Resource footprints and their ecosystem consequences. Sci. Rep..

[5] Rodrigues ASL (2004). Effectiveness of the global protected area network in representing species diversity. Nature.

[6] Naughton-Treves L, Holland MB, Brandon K (2005). The role of protected areas in conserving biodiversity and sustaining local livelihoods. Annu. Rev. Environ. Resour..

[7] Chen Y, Tang Z, Fang J (2009). Distribution of nature reserves and status of biodiversity protection in China. Biodivers Sci..

[8] Leverington F, Costa KL, Pavese H, Lisle A, Hockings M (2010). A global analysis of protected area management effectiveness. Environ. Manage..

[9] Watson JE, Dudley N, Segan DB, Hockings M (2014). The performance and potential of protected areas. Nature.

[10] Ervin J (2003). Rapid assessment of protected area management effectiveness in four countries. BioScience.

[11] Geldmann J, Joppa LN, Burgess ND (2014). Mapping change in human pressure globally on land and within protected areas. Conserv. Biol..

[12] Williams PH (2003). Integrating biodiversity priorities with conflicting socio-economic values in the Guinean–Congolian forest region. Biodivers. Conserv..

[13] Zhang L, Ouyang Z, Xu W (2015). Theory’s work frame and hot issues of systematic conservation planning. Acta Ecologica Sinica.

[14] Margules CR, Pressey RL (2000). Systematic conservation planning. Nature.

[15] Wu, Z. *Flora of Yunnan* (Science Press, 1995).

[16] Yang Y, Tian K, Hao J, Pei S, Yang Y (2004). Biodiversity and biodiversity conservation in Yunnan, China. Biodivers. Conserv..

[17] Yunnan Environmental Protection Department (YEPD). Yunnan biodiversity conservation strategy and action plan 2012–2030. http://www.ynepb.gov.cn/zwxx/zfwj/yhf/201306/t20130608_39091.html (2013).

[18] Minstry of Environmental Protection of the People’s Republic of China (MEPPRC) & Chinese Academy of Science(CAS). China’s Biodiversity Redlist: Volumn of Higher Plant-Appraisal Report. http://www.zhb.gov.cn/gkml/hbb/bgg/201309/W020130912562095920726.pdf (2013).

[19] Minstry of Environmental Protection of the People’s Republic of China (MEPPRC) & Chinese Academy of Science (CAS). China’s Biodiversity Redlist: Volumn of Vertebrates -Appraisal Report. http://www.zhb.gov.cn/gkml/hbb/bgg/201505/W020150525496758954804.pdf (2015).

[20] Luan X (2011). Preliminary assessment on pressure and threat of protected area in northeast China. Journal of Natural Resources.

[21] Vasconcelos RP, Batista MI, Henriques S (2017). Current limitations of global conservation to protect higher vulnerability and lower resilience fish species. Sci. Rep..

[22] Wu R (2011). Effectiveness of China’s nature reserves in representing ecological diversity. Front. Ecol. Environ..

[23] Lin S (2016). Identifying local-scale wilderness for on-ground conservation actions within a global biodiversity hotspot. Sci. Rep..

[24] Xu WG (2016). Distribution of community residents in nature reserves and its impacts on the reserves in China. Journal of Ecology and Rural Environment.

[25] Zheng YM, Zhang HY, Niu ZG, Gong P (2012). Protection efficacy of national wetland reserves in China. Chin. Sci. Bull..

[26] Hockings M (2003). Systems for assessing the effectiveness of management in protected areas. BioScience.

[27] DeFries R, Hansen A, Newton AC, Hansen MC (2005). Increasing isolation of protected areas in tropical forests over the past twenty years. Ecol. Appl..

[28] Jiang LJ, Miao H, Ouyang ZY (2006). An investigation of factors that influence the effects of management of protected areas. Acta Ecologica Sinica.

[29] Yan Y, Wang Z, Gao J, Xu W, Jiang M (2010). Regional distribution characteristics of nature reserves and the influencing factorsin China. Acta Ecologica Sinica.

[30] Quan J, Ouyang Z, Xu W, Miao H (2011). Assessment of the effectiveness of nature reserve management in China. Biodivers. Conserv..

[31] Zhang MG (2012). Using species distribution modeling to improve conservation and land use planning of Yunnan, China. Biol. Conserv..

[32] Cao H, Tang M, Deng H, Dong R (2014). Analysis of management effectiveness of natural reserves in Yunnan province, China. International Journal of Sustainable Development & World Ecology.

[33] Chapin FS (2000). Consequences of changing biodiversity. Nature.

[34] Pimm SL, Raven P (2000). Biodiversity: extinction by numbers. Nature.

[35] Foley JA (2005). Global consequences of land use. Science.

[36] Xu J (2005). Land-use and land-cover change and farmer vulnerability in Xishuangbanna prefecture in southwestern China. Environ. Manage..

[37] Willson A (2006). Forest conversion and land use change in rural northwest Yunnan, China. Mt. Res. Dev..

[38] Li Z, Fox JM (2012). Mapping rubber tree growth in mainland southeast Asia using time-series MODIS 250 m NDVI and statistical data. Appl. Geogr..

[39] Zomer RJ, Xu J, Wang M, Trabucco A, Li Z (2015). Projected impact of climate change on the effectiveness of the existing protected area network for biodiversity conservation within Yunnan province, China. Biol. Conserv..

[40] Xu J, Wilkes A (2004). Biodiversity impact analysis in northwest Yunnan, southwest China. Biodivers. Conserv..

[41] Zomer RJ (2014). Environmental stratification to model climate change impacts on biodiversity and rubber production in Xishuangbanna, Yunnan, China. Biol. Conserv..

[42] Xu J, Melick DR (2007). Rethinking the effectiveness of public protected areas in southwestern China. Conserv. Biol..

[43] O’Connell-Rodwell CE, Rodwell T, Rice M, Hart LA (2000). Living with the modern conservation paradigm: can agricultural communities co-exist with elephants? A five-year case study in east Caprivi, Namibia. Biol. Conserv..

[44] Cincotta RP, Wisnewski J, Engelman R (2000). Human population in the biodiversity hotspots. Nature.

[45] McKee JK, Sciulli PW, Fooce CD, Waite TA (2003). Forecasting global biodiversity threats associated with human population growth. Biol. Conserv..

[46] Luck GW (2007). A review of the relationships between human population density and biodiversity. Biol. Rev..

[47] Ellis EC, Ramankutty N (2008). Putting people in the map: anthropogenic biomes of the world. Front. Ecol. Environ..

[48] Brashares JS, Arcese P, Sam MK (2001). Human demography and reserve size predict wildlife extinction in westAfrica. Proc. R. Soc. Lond. B Biol. Sci..

[49] Rogers HM, Glew L, Honzák M, Hudson MD (2010). Prioritizing key biodiversity areas in Madagascar by including data on human pressure and ecosystem services. Landsc. Urban Plan..

[50] Statistical Bureau of Yunnan Province (SBYP). *2011 Yunnan statistical yearbook* (China Statistics Press, 2011).

[51] Venter O (2016). Sixteen years of change in the global terrestrial human footprint and implications for biodiversity conservation. Nat. Commun..

[52] Kallimanis AS, Panitsa M, Dimopoulos P (2017). Quality of non-expert citizen science data collected for habitat type conservation status assessment inNatura 2000 protected areas. Sci. Rep..

