# Supplementary material for: Human Pressures on Natural Reserves in Yunnan Province and Management Implications
Source: Sci Rep. 2018 Feb 19;8:3260. doi: 10.1038/s41598-018-21654-w (PMC5818542; doi:10.1038/s41598-018-21654-w)
Supplement: Supplementary file 1 — Supplementary Tables [file 41598_2018_21654_MOESM1_ESM.pdf]

Human Pressures on Natural Reserves in Yunnan Province and Management Implications

Cheng Qiu[1,2,3], Jinming Hu[1,3,4*], Feiling Yang[1,3*], Feng Liu[1,3] & Xinwang Li[1,3]

[1]Institute of International Rivers and Eco-security, Yunnan University, Kunming, Yunnan 650500, China. [2]School of Geography and Land Engineering, Yuxi Normal University, Yuxi, Yunnan 653100, China.[3]Yunnan Key Laboratory of International Rivers and Transboundary Ecosecurity, Yunnan University, Kunming, Yunnan 650091, China.[4]Collaborative Innovation Center for Territorial Sovereignty and Maritime Rights, Wuhan, Hubei 430072, China

[*]Correspondence and requests for materials should be addressed to J.H. (email: hujm@ynu.edu.cn) or F. Y. (flyang@ynu.edu.cn)

Supplementary Tables

Supplementary Table S1. The orders of NRs' ICHPI and ECHPI in Yunnan Province

Supplementary Table S2. Internal and external HPI classes of 58 NRs in Yunnan Province

Natural reserves' name	ICHPI	ICHPI grade	ICHPI rank	ECHPI	ECHPI grade	ECHPI rank	Type	Grade
Bita Lake	0.0029	slight	1	0.0146	slight	1	wetland	provincial
Yongde Snow Mountain	0.0335	slight	2	0.6642	light	25	forest	national
Weiyuan River	0.0340	slight	3	0.6151	light	22	plant	provincial
Baima Snow Mountain	0.0380	slight	4	0.1308	slight	2	animal	national
Gaoligong Mountain	0.0422	slight	5	0.3909	slight	11	forest	national
Yunlong Tianchi Lake	0.0466	slight	6	0.2836	slight	5	animal	national
Laiyang River	0.0476	slight	7	0.3007	slight	6	forest	provincial
Wuliang Mountain	0.0665	slight	8	0.6614	light	24	animal	national
Diaoling Mountain	0.0670	slight	9	0.3406	slight	9	forest	provincial
Nangun River	0.0791	slight	10	0.3967	slight	13	animal	national
Laojun Mountain	0.0960	slight	11	0.5964	light	21	forest	provincial
Yulong Snow Mountain	0.1142	slight	12	0.2277	slight	4	forest	provincial
Ailao Mountain	0.1294	slight	13	0.6661	light	26	forest	national
Tongbiguan	0.1421	slight	14	0.6829	light	28	forest	provincial
Huanglian Mountain	0.1491	slight	15	0.3835	slight	10	forest	national
Longling Xiaoheishan	0.1621	slight	16	1.1143	moderate	44	forest	provincial
Dawei Mountain	0.1731	slight	17	0.4851	light	16	forest	national
Nanpeng River	0.1802	slight	18	0.7666	light	31	forest	provincial
Haba Snow Mountain	0.1841	slight	19	0.3293	slight	8	forest	provincial
Lugu Lake	0.2077	slight	20	0.1739	slight	3	wetland	provincial
Yongping Golden Temple	0.2082	slight	21	0.4248	slight	14	forest	provincial
Jiaozi Snow Mountain	0.2413	light	22	0.5830	light	20	forest	national
Xishuangbanna	0.2484	light	23	0.6763	light	27	forest	national
Wumeng Mountain	0.2551	light	24	0.8465	light	34	forest	national

Tuoniang River	0.2696	light	25	0.4639	slight	15	forest	provincial
Lancang River	0.3192	light	26	0.9047	moderate	35	forest	provincial
Yunling	0.3315	light	27	0.3923	slight	12	forest	provincial
Jinping Fenshuiling	0.3367	light	28	0.5006	light	17	forest	national
Shibalian Mountain	0.3500	light	29	1.0108	moderate	39	forest	provincial
Lao Mountain	0.3665	light	30	0.6993	light	30	forest	provincial
Nuozhadu	0.3887	light	31	0.6420	light	23	forest	provincial
Napa Lake	0.4641	light	32	3.0785	severe	55	wetland	provincial
Yuan River	0.4755	light	33	1.0831	moderate	41	forest	national
Weishan Qinghua Green Peafowl	0.5366	moderate	34	1.0867	moderate	43	animal	provincial
Naban River	0.5454	moderate	35	0.3175	slight	7	forest	national
Honghe Amu Mountain	0.5697	moderate	36	1.0516	moderate	40	forest	provincial
Zixi Mountain	0.5994	moderate	37	0.8416	light	32	forest	provincial
Wenshan	0.6629	moderate	38	1.0863	moderate	42	forest	national
Gulinqing	0.7001	moderate	39	0.5326	light	19	forest	provincial
Zhanyi Haifeng	0.7579	moderate	40	0.9745	moderate	38	wetland	provincial
Guanyin Mountain	0.7703	moderate	41	1.2595	moderate	47	forest	provincial
Mojiang Xiqi *Alsophila spinulosa*	0.8022	moderate	42	0.8450	light	33	plant	provincial
Yao Mountain	0.9430	moderate	43	1.2215	moderate	46	forest	national
Menglian Mountain	1.1547	severe	44	0.5319	light	18	plant	provincial
Xundian Black-necked Cranes	1.2255	severe	45	0.9320	moderate	36	animal	provincial
Jiache	1.2397	severe	46	1.1738	moderate	45	plant	provincial
Pear River’s Source	1.2618	severe	47	2.4160	severe	52	forest	provincial
Jian Lake	1.2651	severe	48	2.6379	severe	53	wetland	provincial
Huize Black-necked Cranes	1.3993	severe	49	2.2034	severe	51	animal	national

Cang Mountain and Erhai Lake	1.4822	severe	50	8.6958	extreme	57	wetland/forest	national
Qiubei Puzhehei	1.5400	severe	51	2.0021	severe	50	wetland	provincial
Guangnan Babao	1.5490	severe	52	0.6936	light	29	geological relicts	provincial
Dashanbao	1.8659	severe	53	1.7493	moderate	48	wetland	national
Chengjiang Fossil Site	1.9132	severe	54	2.9938	severe	54	geological relicts	provincial
Lashi Lake	2.6893	severe	55	3.1752	severe	56	wetland	provincial
Jianshui Swallow Cave	3.4382	extreme	56	1.8930	severe	49	animal	provincial
Jinning Meishucun	4.2177	extreme	57	9.8617	extreme	58	geological relicts	provincial
Tengchong Beihai Wetland	5.1281	extreme	58	0.9432	moderate	37	wetland	provincial

Supplementary Table S1. The orders of NRs' ICHPI and ECHPI in Yunnan Province

internal/external	HPI levels	PDPI	GDPI	HLUPI	CHPI
Internal	slight	0.0000~0.1378	0.0000~0.2293	0.0000~0.3308	0.0000~0.2082
	light	0.1379~0.4003	0.2294~0.5290	0.3309~0.7569	0.2083~0.4756
	moderate	0.4004~1.1784	0.5291~2.1974	0.7570~1.2676	0.4757~0.9431
	severe	1.1785~2.3513	2.1975~4.0601	1.2677~2.0572	0.9431~2.6893
	extreme	2.3514~3.6724	4.0602~10.8282	2.0573~3.7653	2.6894~5.1281
external	slight	0.0000~0.5533	0.0000~0.5907	0.0000~0.5833	0.0000~0.4623
	light	0.5534~1.1486	0.5908~1.9579	0.5834~1.0645	0.4624~0.8446
	moderate	1.1487~2.2887	1.9580~4.1735	1.0646~1.6537	0.8447~1.7460
	severe	2.2888~5.3133	4.1736~7.1491	1.6538~2.3869	1.7461~3.1730
	extreme	5.3134~18.3776	7.1492~17.3933	2.3870~3.4829	3.1731~9.8575

Supplementary Table S2. Internal and external HPI classes of 58 NRs in Yunnan Province
